# Digital Maturity Consulting and Strategizing to Optimize Services: Overview

**DOI:** 10.2196/37545

**Published:** 2023-01-17

**Authors:** Peter Phiri, Heitor Cavalini, Suchith Shetty, Gayathri Delanerolle

**Affiliations:** 1 School of Psychology University of Southampton Faculty of Environmental and Life Sciences Southampton United Kingdom; 2 Research & Innovation Department Southern Health NHS Foundation Trust Southampton United Kingdom; 3 Nuffield Department of Primary Care Health Sciences University of Oxford Oxford United Kingdom

**Keywords:** digital maturity model, health care system, electronic medical records, health record, information, UK, medical service, care provider, integration, interoperability, digital health, digital record, workflow

## Abstract

The National Health Service (NHS), the health care system of the United Kingdom, is one of the largest health care entities in the world and has been successfully serving the UK population for decades. The NHS is also the fourth-largest employer globally. True to its reputation, some of the most modern and technically advanced medical services are available in the United Kingdom. However, between the acute, primary, secondary, and tertiary care providers of the NHS, there needs to be seamless integration and interoperability to provide timely holistic care to patients at a national level. Various efforts have been taken and programs launched since 2002 to achieve digital transformation in the NHS but with partial success rates. As it is important to understand a problem before trying to solve it, in this paper, we focus on tools used to assess the digital maturity of NHS trusts and organizations. Additionally, we aim to present the impact of ongoing transformation attempts on secondary services, particularly mental health. This paper considered the literature on digital maturity and performed a rapid review of currently available tools to measure digital maturity. We have performed a multivocal literature review that included white papers and web-based documents in addition to peer-reviewed literature. Further, the paper also provides a perspective of the ground reality from a mental health service provider’s point of view. Assessment tools adopted from the global market, later modified and tailor-made to suit local preferences, are currently being used. However, there is a need for a robust framework that assesses status, allows target setting, and tracks progress across diverse providers.

## Introduction

### Overview

The National Health Service (NHS) in the United Kingdom accounts for 3% of the global health market [1]. The NHS has stayed committed to its founding health care principles of universality, free at the point of delivery, and equity, and is paid for by central funding. It remains the fourth largest employer in the world, meeting the needs of a growing and complex population in the United Kingdom.

The NHS is also a world-renowned entity for conducting high-quality clinical research and partnering with industry and academics alike. Hence, its ability to continuously improve and promote efficiency is vital for remaining fit for purpose. Part of this aspect is the need to harness the power of advancements made in health care technologies. This requires continuous improvements in the technological literacy of both health care professionals and patients.

### Digital Transformation of NHS at Scale

Independently and locally, a few NHS trusts have implemented advanced IT solutions. The NHS is among the leading users of IT in health care in the world. The United Kingdom was ranked third in the world in the science and technology division of the World Index of Health Innovation ranking of 2021 [1]. However, the balance of power and decision-making in the NHS is led locally and not centrally controlled. There were two expensive centralized attempts made in 2002 and 2011, both of which failed to reach the primary objective of delivering an electronic health record (EHR) system that contains patient records from across the United Kingdom. The National Programme for IT [2] was launched in 2002 with a 10-year reform plan and a primary objective of building a national system such that a patient’s health record is readily available to whoever is responsible for the patient’s care. The program delivered a few successful aspects, which continue to be in use to date, but could not achieve its primary objective. With growing demand and resourcing costs as well as changes in the economic growth in the United Kingdom, NHS operational costs for IT infrastructure continue to grow.

While there are challenges across global health care systems to adopt technologies due to various factors such as resources, infrastructure, and costs, the mental health care organizations, in particular, have seen these to be the furthest behind in comparison to acute care [3,4]. Evidently, an equal budget is not allocated to mental health care in comparison to other areas. Mental health problems contributed to 23% of the total burden in the United Kingdom but were only allotted 14.8% of the health budget in 2021 and 2022 [5]. The remanences of fragmented approaches between acute and primary care still require addressing although there are vast IT infrastructural changes that have incurred over the last decade [6]. A contributory factor could be multiple hierarchical boards making decisions that are not necessarily aligned with the national digital strategy for health care. A good example of this has been delays associated with the national “NHS paperless” drive that was established in 2014 with a completion timeline of 2018, which was then postponed to 2020 and has not been achieved quite yet. An independent report [7] published in September 2016 by the National Advisory Group on Health Information Technology in England suggested that all trusts should aim to be largely digitized by 2023 rather than 2020. Another independent research report [8] from April 2017 by Digital Health Intelligence concluded that the “paperless” target would not be met before 2027. The NHS needed to change its strategy toward achieving digital transformation. The redefined target now is to reach a “core level of digitisation” by 2024 or 2025. NHSX, a joint unit between the Department of Health and Social Care, NHS England, and NHS Improvement (NHSE&I), was hence launched in July 2019 with the responsibility of setting national policies and developing best practices for the NHS through digital transformation. Three years later, NHSX has been merged into NHS England, along with NHS Digital, Health Education England (HEE) [9].

## Method: Digital Maturity

Digital maturity is an important facet to consider at this point given it measures an organization’s ability to develop value-based models through digitizing key tasks to better manage, predict, and sustain transformation from a clinical service and business provision perspective. Digital maturity is an indicator of the capability and compatibility of the IT systems to interface and interact both within and across organizations. However, it is vital to use a standard digital maturity assessment (DMA) across NHS foundation trusts, district general hospitals, and community hospitals to understand the current IT infrastructure versus the requirements for the population they serve and budgetary allocations provided. The digital maturity of organizations are evaluated using frameworks like DMAs. In the next section, we present some of the widely used DMA tools.

## Assessment of Digital Maturity

### Electronic Medical Record Adoption Model

One of the most widely used assessment tools to evaluate the digital maturity of health care providers is the Electronic Medical Record Adoption Model (EMRAM) [10]. EMRAM is a flagship maturity model developed by Healthcare Information and Management Systems Society (HIMSS), an US-based nonprofit organization. EMRAM offers organizations a prescriptive framework with a vendor-neutral road map to build digital health ecosystems that are in line with a global benchmark. In addition to electronic medical record (EMR), HIMSS now offers separate maturity models for analytics (Adoption Model for Analytics Maturity), continuity of care (Continuity of Care Maturity Model [CCMM]), clinical supply chain (Clinically Integrated Supply Outcomes Model [CISOM]), digital imaging (Digital Imaging Adoption Model), infrastructure (Infrastructure Adoption Model [INFRAM]), and outpatient EMR (Outpatient Electronic Medical Record Adoption Model). These models are intended to aid regulatory bodies and health care providers to deliver lasting improvements in their care outcomes. All these models have levels/stages from 0 to 7 where a hospital is placed as per its assessment score. The levels range from stage 0 where the three key ancillary department systems (ie, laboratory, pharmacy, and radiology) are not installed to stage 7 where the hospital has complete EMRs with no paper being used to manage and deliver patient care.

In Europe, around 2500 hospitals have undergone the EMRAM assessment, with HIMSS stating that there is much opportunity for advancement and maturity. The latest reports from EMRAM Analytics [11] show 3 NHS hospitals at stage 7 and 4 hospitals at stage 6. Cambridge University Hospitals (CUH) [12] NHS Foundation Trust became the first in the United Kingdom to reach EMRAM stage 7 in October 2020. While the transition from paper to digital was initiated in 2014 at CUH, it was at stage 6 of EMRAM validation in November 2015 and attained stage 7 when re-evaluated in October 2020. Consider the example of Salford Royal NHS Foundation Trust [13], which was placed at level 5 when they began the EMRAM assessment in 2015. Though there was a target to reach level 7 within 12-19 months, there has been no announcement on a re-evaluation or a follow-up in 6 years. It is not a common feat for hospitals to reach EMRAM stage 7. Of 5487 registered US hospitals in 2017, only 6.4% [14] have attained EMRAM stage 7 status and ~33% each were at stages 5 or 6. The HIMSS Analytics [7] report shows a total of 257 hospitals worldwide with stage 7 status, of which 209 are in the United States. Saudi Arabia is second on the list with 17 stage 7 hospitals.

There are limitations [15], nonetheless, to the EMRAM assessment that limits its wider application to the health care setting in the United Kingdom in a way that can aid policy decisions for digital transformation. Being technology focused, this assessment gives more weightage to functionality. Driving transformation by enabling staff to embrace and adapt lacks attention. The right infrastructure needs to be combined with an operational drive from the root level, which can only occur when the framework is cost-effective, flexible, and user-friendly. The success of technology-supported programs within health and social care is not limited to functional implementation, as they can fail due to nonadoption or abandonment by individuals, fail to scale up locally or spread distantly, or fail to sustain over the long-term [16]. Moreover, the scope of the assessment is narrow and localized. While it evaluates sharing of information within a hospital, it leaves out other health care organizations like primary and secondary care that together deliver integrated care to the patients. For example, with over 7000 diabetes-related leg amputations reported per year in the United Kingdom, a robust foot care pathway is of urgent need. A concise pathway with a complete loop must be in place with clear communication across clinicians and health care workers in the primary care team, foot protection service, and secondary care.

In another example, the origins of patients seeking mental services are in primary care, and they are referred to secondary care mental health providers only when their condition worsens. This can be avoided if digital services are used to remove the disconnect between the two service providers. Efficient care pathways ensure that patients get a timely diagnosis, and health care workers can efficiently share the care to guarantee safe and effective treatment. In the case of cancer care pathways, for instance, there is a waiting target time set for the diagnosis and start of treatment in the United Kingdom. A patient should not wait more than 28 days from referral to diagnosis, and there should not be more than 62 days of wait between an urgent referral and the start of treatment. In the case of obstetrics, the NHS assesses the quality of care provided during antenatal and postnatal phases by relying on surveys of women’s experience of care. Survey results of 2019 report that most women had a positive experience of maternity care with possible improvements in the continuity of carer, postnatal care, and perinatal mental health area. EMRAM fails to capture this aspect of care pathways across health care sectors.

To measure progress toward digital transformation, HIMSS has a broader framework called the digital health indicator (DHI). The DHI assessment is layered across 4 components, namely, governance and workforce, interoperability, analytics and traceability, and digital capacity leading to consumer-enabled health. Though HIMSS offers a host of other assessment tools like the INFRAM, CISOM, CCMM, etc, through the DHI framework, their penetration into the global market is lower.

### NHS England Digital Maturity Index

NHS England has combined components of EMRAM with aspects that assess infrastructure, interoperability, readiness, etc, to come up with its version of a Digital Maturity Index [17]. The DMA model developed by NHS England and NHSE&I is a self-assessment questionnaire with 175 questions to be answered by the local trusts and organizations. Questions are aggregated to form 3 themes:

1. Readiness: 30 questions focused on strategy alignment, leadership, resourcing, governance, and information governance to evaluate the ability to plan and deploy digital services
2. Capability: 134 questions focusing on the extent to which technology is currently being used to deliver care, including questions that cover records, assessments and plans, orders and results, management, transfers of care, medicines optimization, decision support, remote and assistive care, business and clinical intelligence, standards, asset and resource optimization, etc
3. Infrastructure: 15 questions to assess the quality of underlying IT systems, focusing on aspects like the availability of Wi-Fi networks, hardware performance, provisions for business continuity/disaster recovery, etc

An NHS trust can rate itself for each question as low (<40%), medium (40%-69%), or high (≥70%). The assessment questionnaire developed in 2016 was last revised in 2017 by tailoring the questions to suit acute, mental health, community, and ambulance organizations. There is a separate self-assessment questionnaire by NHSE&I for general practitioners (GPs). The Local Government Association relies on an assessment tool similar to that of NHSE&I to gauge the digital maturity of local authorities that carry out social care responsibilities.

In 2017, self-assessment reports of digital maturity from trusts across the NHS revealed grim statistics. Only 54% of trusts reported that their staff could rely on digital records, while 16% of trusts’ IT systems were Systematized Nomenclature of Medicine Clinical Terms (SNOMED CT) compliant, which is an international standard for clinical terminology, and 16% of trusts reported themselves to be low in their capability to use digital technology to deliver care. It is to be noted that self-assessed scores reported by the trusts are not all validated by NHSE&I. Only a sample number of trusts that marked themselves “high” were validated by NHSE&I. Since the current assessment model is self-based and not peer-based, there is also the possibility of trusts and organizations gaming the system by understanding the consequences of their self-reported assessment scores to, for example, get more funding. For instance, in the 2017 report, while 77% of the trusts completely agreed that they participated in initiatives across the health and care community to achieve digital record sharing, only 48% completely agreed that digital technology was used to support improved collaboration and coordination across different parts within their organization. Table 1 compares the advantages and disadvantages of the NHS’s DMA tool and EMRAM tool from HIMSS.

Owing to the limitations and lack of agility of the current maturity assessment models, members of the Global Digital Exemplar program’s independent evaluation team proposed an alternative framework that measures digital maturity considering the existing institutional and technological infrastructure and is tailored to the needs of the local population [18].

**Table 1:** Advantages and disadvantages of National Health Services’ (NHS) digital maturity assessment (DMA) tool and the Electronic Medical Record Assessment Model (EMRAM) tool from Healthcare Information and Management Systems Society (HIMSS).

DMA tool	Advantages	Disadvantages
EMRAM from HIMSS	Globally recognized standard Prescriptive framework Peer-reviewed and evaluated	Penetration is currently limited to the United States and Canada Heavily technology focused, with limited weightage to people and process Limited to hospital settings
Digital Maturity Index of NHS England	Customized for NHS England Broader in scope	Difficult to compare with Global benchmarks Self-reported assessments with limited validation

## Factors Affecting Digital Maturity

### People: Digital Skills of the Workforce

Infrastructure and processes aside, the success of a transformation drive depends on the willingness and capability of end users to adopt and adapt to the change. The UK population, both young and old, is largely technologically literate although the disparity between those who have access to the internet varies. To understand the areas of learning needed so that effective and efficient training can be provided, HEE is working on a digital literacy self-assessment tool [19] through its Digital Readiness program. This web-based digital diagnostic tool helps assess the digital skills of the wider workforce by letting them determine their current digital literacy levels. The tool is currently in the beta-testing phase and is expected to be rolled out to the wider health and social care workforce by spring 2022. However, less than 1% [20] of the 2020-2021 Digital Transformation Portfolio budget has been allocated to “Building a Digital-Ready Workforce.” Since professional behavior generally tends to change slower than consumer behavior, it is important to do more to engage the staff and help them gain digital skills. A survey [21] result showed patients, in general, are more enthusiastic about technology as it provides greater autonomy in choosing health care options. A systematic review by Virtenan et al [21] identified 29 different behavior change techniques to promote the technical competency of health care professionals but noted that the techniques are more focused on capability than motivation. While motivation varies based on the subjective perspective of value, urgency, risk, and compatibility, it is promising to see growing interest among health care workers to enhance their digital skills when support is provided. This is evident from the changing use trend of the eLearning program offered by HEE. When access to HEE’s eLearning platform was made free for learners from the social care sector in light of the COVID-19 pandemic, the use count increased from 500,000 sessions per month to 200,000 sessions per day. Additionally, remote GP consultations saw a substantial increase during the pandemic period from 90% of consultations being face to face in 2019 to 85% being remote consultations on the phone or via video during the peak of the pandemic.

There are multiple programs launched by the NHS to promote the adoption of innovation and technology. The Innovation and Technology Payment program helps remove financial and procurement barriers to introducing new technology. Rapid Uptake Products promotes stronger adoption of innovative products with lower-than-expected uptake to date. Through its Digital Academy, the NHS hopes to generate excellent leaders and drivers of digital transformation. Code4Health [22] is a program launched to help clinical staff gain digital skills like developing apps or other digital services. For example, in collaboration with a senior clinician, an NLP-enabled mental health chatbot was developed through Code4Health that can help with the triage of a new patient seeking mental health services. The tool is still under development and is not available for wide-scale deployment for live patient assessments. However, such tools when widely used have the potential to alleviate the demand-supply bottleneck currently being experienced in areas like mental health services.

On the other hand, with more dependence on technology, there needs to be a robust IT support system, as any form of technology failure can seriously disrupt patient care. Currently, with 4% to 5% of the NHS workforce holding roles in informatics, there is a shortage of digital and data skills. The profession of informatics is not widely recognized within NHS [23], while these skills have a global competitive market. There is a need for a systematic plan to develop informatics as a profession within the NHS.

### Infrastructure: The National Standard for IT Systems

The EHR or EMR systems used by trusts across the country need to have a common standard to ensure compatible sharing of data and information as and when needed. Without interoperability, sharing of data across systems becomes challenging. Currently, there are three options [17] that the NHSX has offered the trusts to choose from: buying an enterprisewide system, integrating multiple record systems, or building a compatible native system. Of these, trusts with enterprisewide systems appear to have higher digital maturity, but this option comes with a higher upfront cost. However, there is no robust estimate of the whole-life-cost of the three options that can help trusts make an informed decision.

Apart from being used for the direct care of the patients, the data collected in EHRs are also used for medical research and other secondary purposes like service evaluation and planning. However, the public has the right to opt out of their data being used for secondary purposes. Currently, only 3% of patients chose to opt out. The larger the volume of data, the better it is for secondary purposes. So, it is important to keep the data secure against possible cyberattacks and maintain public trust.

### IT System in Primary Care

The general medical service contracts fund the core IT systems of all GPs. This central system gives far greater control to the policy makers in GP systems than in hospital systems. However, studies [24] report that the choice of a clinical computer system in primary care appears to be geographically clustered. While clustering suppresses choice, systems that are not interoperable pose barriers to accessing data stored in other systems. The GP Systems of Choice (GPSoC), a contractual framework to supply IT systems and services to GP practices, allowed only 4 IT systems to choose from, namely, Egton Medical Information Services (EMIS), The Phoenix Partnership (TPP), Microtest, and In Practice Systems. Currently, 95% of the IT systems of GPs are supplied by 2 suppliers, EMIS and TPP. Through an interoperability project, TPP’s SystemOne and EMIS Health have enabled data sharing between the two systems since 2019 [25], but the project is still a work in progress. GPSoC was replaced by NHS Digital in 2019 by the GP IT Futures framework. To promote innovation and competition, and to develop the GP IT market, the new framework allows GPs to choose their IT system from 69 suppliers. However, the new framework with more supplies and systems is likely to complicate interoperability. Within primary care, practice variability is significant between those in rural areas and metropolitan cities and towns. In addition, primary care general practices have varying scalability based on the income they receive which in turn is based upon the number of patients registered. Therefore the IT infrastructure requirement of a large practice would differ from that of a practice with a smaller number of patients. This variability challenges the unification of the findings of the DMA.

### IT System in Secondary Care

In recent years, integrated care systems have been developed through partnerships between primary and secondary care organizations with a view to combine the management of physical and mental health, improve population health, and minimize health care inequalities. A key system for this purpose is the patient record system. There are various providers although Cerner Millennium appears to be a common system that is Java and cloud based. This system supports the maintenance of long-term health care records in a secure manner. However, there are acute care–specific systems based on clinical specialty needs such as the Picture Archiving Communication System (PACS) that provides reports and images of radiological examinations. These are stored and reported digitally in a secure manner. Some NHS organizations also use cloud-based systems to support PACS.

## Discussion

The digital maturity of the NHS has improved with the establishment of various programs as demonstrated by the independent report published by the National Audit Office in May 2020 [26]. With increasing clinical demands that require advancement within the IT infrastructure because of a growing population with complex clinical needs and the COVID-19 pandemic, the impact on the current digital maturity proportions across the NHS has substantially changed. The NHS had identified the importance of seamless data sharing between the IT systems for their patients and the need for a national standard as a prerequisite as early as 1998. However, the implementation has turned out to be quite challenging. It is imperative to stabilize the growing demand for IT infrastructure with an established governance framework underpinned by legislative measures that align with the national standard.

Increasingly, the health support systems are concerned about introducing themselves to the technology. The NHS, for instance, has worked to implement the technology in all areas of health care. To improve the intercommunication between systems, the NHS has implemented a digital maturity. In addition, DMAs can offer a pragmatic method to establish a “roadmap” or inform a path, and this enables organizations to understand and engage their staff meaningfully to challenge existing workflows with digital technology [27]. However, this is not an easy task, as the substantial amount of data to be introduced into the system, added to the high demand for specialized training to master the system, can be major barriers to the transition to a more modern system [28].

Health and social care operate as separate independent systems managed in silos though they are required to work hand in hand to deal with multiple, long, and complex care requirements of patients. This separation has complicated coordinating care due to difficulties with data sharing [29]. Despite multiple reports [30] raising the issue and calling for an effort to further integrate health and social care, progress over the years has been slow. Social care is commissioned and provided by local authorities through mainly private providers. For example, the Delayed Transfer of Care report shows that one-third of the delay in transferring patients ready to leave the hospital is due to the unavailability of social care services [31].

### Conclusion

The various components that together make up the health and care system in the United Kingdom are vastly diverse and complex. After the failed attempt by an overly centralized approach to digitize secondary care at a national level and repeated delays in reaching “paperless” status, policy makers are now working on the more realistic albeit challenging task of achieving “a core level of digitization” by 2024/2025. Programs and policies are touching upon various pieces of the puzzle that can come together to help achieve the target objective. People, process, and technology infrastructure are the three pillars that need equal focus and attention to achieve success in an initiative like digital transformation. All three factors are receiving their due attention in the renewed plan, which is promising. As with any problem-solving exercise, the first step is to get a thorough understanding of the problem itself. DMA tools are used for this purpose. Direct adoption of a prominent tool used internationally has its limitation, as it does not cater to the local nuances and idiosyncrasies. Building on top of such a tool that is considered a reliable benchmark, the NHS is currently relying on its tailor-made version. In our opinion, the current assessment framework is limited in its scope, and it is vital to have a robust framework that assesses the status and continually tracks the progress of all participating trusts and organizations to boost the overall digital maturity of the NHS.

## Abbreviations

CCMM: Continuity of Care Maturity Model
CISOM: Clinically Integrated Supply Outcomes Model
CUH: Cambridge University Hospitals
DHI: digital health indicator
DMA: digital maturity assessment
EHR: electronic health record
EMIS: Egton Medical Information Services
EMR: electronic medical record
EMRAM: Electronic Medical Record Adoption Model
GP: general practitioner
GPSoC: GP Systems of Choice
HEE: Health Education England
HIMSS: Healthcare Information and Management Systems Society
INFRAM: Infrastructure Adoption Model
NHS: National Health Service
NHSE&I: NHS Improvement
PACS: Picture Archiving Communication System
SNOMED CT: Systematized Nomenclature of Medicine Clinical Terms
TPP: The Phoenix Partnership
